# Screening of Bacteria Inhibiting *Clostridium perfringens* and Assessment of Their Beneficial Effects In Vitro and In Vivo with Whole Genome Sequencing Analysis

**DOI:** 10.3390/microorganisms10102056

**Published:** 2022-10-18

**Authors:** Zipeng Jiang, Weifa Su, Mingzhi Yang, Wentao Li, Tao Gong, Yu Zhang, Chaoyue Wen, Xinxia Wang, Yizhen Wang, Mingliang Jin, Zeqing Lu

**Affiliations:** 1 National Engineering Research Centre for Green Feed and Healthy Farming, Zhejiang University, Hangzhou 310058, China; 2 Key Laboratory of Molecular Nutrition, Ministry of Education, Zhejiang University, Hangzhou 310058, China; 3 Key Laboratory of Animal Nutrition and Feed, Ministry of Agriculture and Rural Affairs, Zhejiang University, Hangzhou 310058, China; 4 Key Laboratory of Animal Nutrition and Feed Nutrition of Zhejiang Province, Zhejiang University, Hangzhou 310058, China; 5 Institute of Feed Science, College of Animal Science, Zhejiang University, Hangzhou 310058, China

**Keywords:** JinHua pig, probiotics, whole genome sequencing, mice model, immunity, gut microbiota

## Abstract

Various countries and organizations call for banning the use of antibiotic growth promoters (AGPs) as prophylaxis and for growth promotion in the livestock industry. Hence, seeking a substitute for antibiotics is strongly required by the livestock industry to maintain the productivity level and profits. Probiotics could represent one viable solution because of their beneficial effects on host health and maintaining the intestinal microbiota balance. In the present study, we aimed to isolate bacterial strains with probiotics properties from JinHua pig (a Chinese native pig breed) gastrointestinal tract that have antagonistic activity against to common disease-causing bacteria on farms. The four most potent strains were isolated (PP31, BA11, BA40, BV5) by the agar well diffusion method and further characterized by acid, bile salt, trypsin tolerance, whole genome sequencing (WGS), and suppressing *Clostridium perfringens* adhesion to IPEC-J2 cells. According to these results, BA40 had the highest number and variety of probiotic secondary metabolic secretion genes and capacity to exclude the attachment of *Clostridium perfringens* to IPEC-J2 cells as same as PB6. The animal experiment in vivo illustrated that BA40 and PB6 could reduce the phenomenon induced by *Clostridium perfringens* challenge of body weight loss, colon length decrease, pro-inflammatory cytokine increase, and *Clostridium perfringens* and *Escherichia coli* increase. The present study provides evidence that BA40 could represent a novel probiotic candidate as PB6, which exhibited some probiotic features and mitigated the burden of *Clostridium perfringens* associated gut disease.

## 1. Introduction

There are many common pathogens that could cause foodborne diseases, such as *Escherichia coli*, *Salmonella enterica*, *Staphylococcus aureus*, and *Clostridium perfringens*, which also cause diseases in animals, including cattle [1], poultry [2], and pigs [3,4]. As for *Clostridium perfringens*, it always involves intestinal problems in animals with a huge economic loss in the livestock industry because of the high mortality rate [5,6]. These animal products with security risks may cause many problems, including an increase in livestock mortality and a decline in production and foodborne illnesses. Using antibiotics can reduce bacterial infections. However, the use of antibiotics for feed purpose is banned and for other purpose is strictly regulated because the spread of antibiotic-resistant pathogens has become a serious problem [7,8]. Therefore, the development of a safe and efficient additive with antimicrobial properties has attracted scholars’ attention. Numerous alternative choices of substitutes emerged, including phytogenic feed additives, antimicrobial peptides, bacteriophages, prebiotics, and probiotics [9,10,11]. Among these alternatives, probiotics represent one viable alternative because of its beneficial effects to host health and maintaining the intestinal microbial balance [12]. Probiotics were defined by World Health Organization (WHO) in 2001—”Probiotics are live microorganisms in sufficient numbers that, when administered, are beneficial to the health of the host” and the International Scientific Association for Probiotics and Prebiotics (ISAPP) strengthen this concept of probiotics [13]. One mechanism for probiotics playing important roles is that it can inhibit the proliferation of pathogenic bacterial and stimulate the growth of beneficial microorganism. Probiotics can interact with gut microbiota and improve the microbial barrier function [14]. Besides, many bacterial genera have been described as probiotic properties with anti-pathogenic activity, such as *bacillus* and *lactobacillus* [15,16,17,18]. Now, some bacterial of the host’s gut community, such as many *bacillus* and *lactobacillus* strains, have been good probiotic candidates.

Generally, native breeds of livestock have higher disease resistance than commercial breeds. PB6 was obtained from health broilers and broadly used for inhibiting *Clostridium perfringens* in the livestock industry [19,20,21,22]. JinHua pigs (a Chinese native pig breed, named China panda pig) have stronger resistance to bacterial invasion than Landrace × Yorkshire (commercial breed) pigs [23]. Hence, screening the isolates from the JinHua pig’s intestinal mucosa as probiotics candidates is a good way to find antibiotic alternatives in the livestock industry. Probiotics can achieve the best effects when they are alive and in a similar environment to where they were isolated from in the same species [24]. Although these probiotics have advantages, we have to evaluate the safety of them and isolate them from the healthy organism. Therefore, we aimed to isolate potential probiotic candidates from the ceca mucosa of finishing JinHua pig, based on anti-pathogenic capacity (especially for *Clostridium perfringens*), acid tolerance, bile salt tolerance, trypsin tolerance, whole genome sequencing, and capacity to exclude the attachment of *Clostridium perfringens* to IPEC-J2 cells. Then we verified the safety and efficacy of the isolated strains in mice model.

## 2. Materials and Methods

We carried out all the procedures in accordance with the university’s regulations on animal experimentation and strictly enforced the guidelines of the Institutional Animal Care and Use Committee of Zhejiang University (ZJU20220164) during the experimental process.

### 2.1. Isolation of Microbes from JinHua Pig Intestines

JinHua pig (Chinese panda pigs, well known for their resistance to disease) feces were collected and sterile swabs (Biosigma Inc., Cona, Italy) were used to collect the fecal samples. Sterile swabs can avoid contamination and the fecal samples were maintained in a solution with 25% (*v*/*v*) glycerol and stored in liquid nitrogen to preserve the microorganisms as the previous study [25].

We mixed 1 g of fecal samples with normal phosphate-buffered saline (PBS) to get suspension. Furthermore, 10-fold serial dilutions by sterilized water were performed in our laboratory. 100 μL of 10-fold dilutions samples was incubated anaerobically in Man, Rogosa, and Sharpe (MRS) plate, and aerobically in Luria Bertani (LB) plate at 37 °C for 24 h till a single colony of appropriate size was grown, respectively. Representative colonies were selected, and the colonies were purified by placing them on new agar plates. The purified colonies were cultured in the corresponding broths and stored at −80 °C with 25% (*v*/*v*) glycerol.

### 2.2. Bacterial Strains Preparation

The following strains were used to detect the antimicrobial activity of probiotics candidates: *Escherichia coli* (ATCC), *Salmonella enterica* (ATCC29628), *Staphylococcus aureus* (ATCC6538), and *Clostridium perfringens* (ATC13124) were purchased through China Center of Industrial Culture Collection (CCICC). *Salmonella enterica*, *Escherichia coli*, and *Staphylococcus aureus* were cultured in LB broth overnight at 37 °C. *Clostridium perfringens* was cultured under an anaerobic environment in Reinforced Clostridium Medium (RCM) for 24 h at 37 °C. The *Bacillus subtills* PB6 (ATCC-PTA 6737) were purchased from Kemin Industries and cultured in LB broth at 37 °C for 12 h. PB6 was used as a positive probiotic control. Isolated strains from JinHua pig feces were cultured in LB broth or RCM broth at 37 °C under aerobic or anaerobic conditions as previous study described [26]. All isolated strains were purified three times since the probiotics were isolated from the intestinal mucosa on agar plates. Then all probiotics candidates were stored at −80 °C.

### 2.3. Preparation of Culture Supernatant and Agar-Well Diffusion Method

We used agar well diffusion to test antimicrobial activity of isolates from JinHua pigs [27,28]. Briefly, the broth (LB or RCM) with 1.5% agar (10 mL) was poured onto the sterile Petri plates (10 × 10 mm). When the agar solidified, we seeded it with 1% of pathogen cultures (approximately 1 × 10^8^ CFU/mL) in autoclaved LB or RCM broth (containing 0.75% agar and cooled about 45 °C) 10 mL. When the plate solidified, 8-mm diameter Oxford Cups were placed on the plate to make five wells, then each well was filled with 100 μL cell-free supernatant (CFS) extracted from isolates. These plates were refrigerated at 4 °C for 4 h. Then the plates were incubated at 37 °C for 12–24 h according to the growth requirements of each pathogen. We obtained CFS by centrifuging at 6000 rpm for 5 min, filtration through a 0.22 μm pore size filter (Millipore, China). Finally, the zone of inhibition could be visibly observed as a clear location where there was no obvious pathogenic growth. The diameters of the inhibition zone were measured by calipers. Each test was carried out in triplicate.

### 2.4. Identification of the Selected Isolates by 16S rRNA Sequencing

16S rRNA analysis method were used to test the isolated probiotic strain candidates First, extracting Genomic DNA by DNeasy Blood and Tissue Kit (Qiagen, Toronto, ON, Canada) as previous study [29]. For isolates, the amplified gene of 16S rRNA using the 27F (5′-AGAGTTTGATCCTGGCTCAG-3′) and 1492R (5′-CTACGGCTACCTTGTTACGA-3′) universal primer sets. Amplifications by PCR and sequenced by the Shanghai Majorbio Bio-Pharm Technology Co.,Ltd (Shanghai, China). The results of sequences were aligned against 16S ribosomal RNA database by using the National Centre for Biotechnology Information (NCBI) BLAST platform. Finally, the sequencing results were uploaded to the GeneBank database: ON227058 for *Bacillus amyloliquefaciens* 40 (BA40), ON227093 for *Bacillus amyloliquefaciens* 11 (BA11), ON227128 for *Bacillus velezensis* 5 (BV5) and ON228197 for *Pediococcus pentosaceus* 31 (PP31).

### 2.5. Growth Characteristics, Acid, Bile Salts and Trypsin Tolerance

Streaking and inoculating PB6, PP31, BA11, BA40, and BV5 onto plates to activate these strains. Briefly, 1% activated strains were anaerobically or aerobically in LB or MRS broth at 37 °C, respectively.

The previous study [30] used the acid, bile, and trypsin to examine the resistance of probiotics candidates. 1% of activated strains was incubated in LB or MRS broth with pH (2.5, 3.5, 4.5), bile salts (0.2%, 0.5%, 0.8%), or trypsin (0.2%, 0.3%, 0.4%). Then, probiotics candidates were incubated at 37 °C for 2 h under anaerobic or aerobic condition (200 rpm shaking). We used the ten-fold serial dilutions method and drop plating method to enumerate the colony counts. The control is 0 h. The survival ratio of probiotics candidates in different pH conditions, different bile salts, or trypsin levels were calculated by the colony counting method, respectively.

### 2.6. Suppress Pathogen Adhesion to IPEC-J2 Cell

IPEC-J2 cells were cultivated using the method in the previous study [31]. Separately cultured isolated strains and *Clostridium perfringens* ATCC13124, and collected the bacteria respectively. Then we washed three times with PBS and resuspended by DMEM/F12 medium. Then the isolated strains were adjusted concentration at 1 × 10^8^ CFU/mL, and *Clostridium perfringens* was adjusted at 1 × 10^6^ CFU/mL. Antibacterial activity experiments were performed by competition, exclusion, and replacement trials, respectively. The co-culture of isolated strains of *Clostridium perfringens* were placed onto the IPEC-J2 cell plate for 2 h, then we tested their competition ability with pathogens. The exclusion trail process was presented as follows: Isolated strains were added onto the IPEC-J2 cell plate and incubated in an atmosphere of 5% CO_2_ at 37 °C, 1 h. Then we discarded the supernatant and washed the plates three times with PBS; subsequently, we added *Clostridium perfringens* to the co-culture for 1 h again to test the exclusion ability. The difference between the replacement and exclusion trails was that the order of lying the isolated strains was different. Briefly, *Clostridium perfringens* and IPEC-J2 cells were co-cultured for 1 h, and other isolated strains were added to the mixture 1 h. Besides, all the control groups used DMEM/F12 to replace isolated strains. Finally, we calculated the inhibition rate via the numbers of live *Clostridium perfringens*. The formula is:

$$\mathrm{Inhibition}\;\mathrm{rate}=\frac{\mathrm{Control}\;\mathrm{group}-\mathrm{Experimental}\;\mathrm{group}}{\mathrm{Control}\;\mathrm{group}}$$

### 2.7. Whole Genome Sequencing and Analysis

We extracted Genomic DNA by SPARKeasy Genome DNA Purification kit (SparkJade, Jinan, China) in accordance with the manufacturer’s instructions. The high purity DNA was sequenced at Shanghai Majorbio Biopharm Technology Co., Ltd. by Illumina sequencing platforms (Hiseq X Ten; Illumina, San Diego, CA, USA), and the method was conducted as the previous study [32]. The low-quality Illumina reads were filtered off to acquire clean data for further analysis. Raw reads quality control was also performed, including base quality, error rate, and distribution. The quality checked DNA samples were constructed with inserts of ~400 bp, and paired-end reads with a length of 150 bp, providing no less than 100× coverage depth of the genome for each sample. Then the Illumina reads were used to estimate the genome size, repeat content, heterozygosity, and finally assembled by SOAPdenovo (Version 2.04 (https://github.com/aquaskyline/SOAPdenovo2 accessed on 24 December 2021)) as previous to generate genome scaffold. Sequenced data were deposited at NCBI GeneBank database under the BioProject ID PRJNA826263, with accession numbers JALMGL000000000 to JALMGO000000000 and accessed on 8 August 2022.

Glimmer (Version 3.02 (http://ccb.jhu.edu/software/glimmer/index.shtml accessed on 24 December 2021)) predicted the coding sequences (CDSs) and Non-Redundant Protein Database (NR), Swiss-Port, Pfam, Gene Ontology (GO), Clusters of orthologous Group (COG), and Kyoto Encyclopedia of Genes and Genomes (KEGG) annotated it. Sequence alignment tools such as antiSMASH (Version 4.0.2 (https://dl.secondarymetabolites.org/releases/4.0.2/ accessed on 24 December 2021)), Virulent Factor Database (VFDB, Version:2016.03 (http://www.mgc.ac.cn/VFs/main.htm accessed on 24 December 2021)), and Comprehensive Antibiotic Resistance Database (CARD, Version 1.1.3 (https://card.mcmaster.ca accessed on 24 December 2021)) were used to classify and predict the gene function and gene annotations were obtained from the best-matched subject (*E*-value < 10^−5^). We used the free online Majorbio Cloud Platform (https://cloud.majorbio.com accessed on 24 December 2021) and analyzed all data.

### 2.8. Animal Experiment

**Figure 1:**
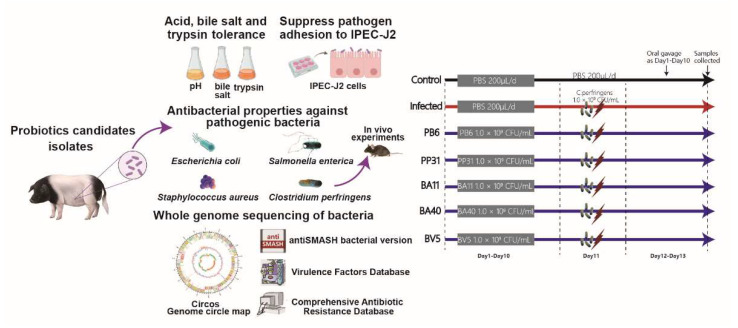
Experimental design.

We purchased forty-two mice (five-week, male C57BL/6) from Shanghai Laboratory Animal Co., Ltd. (SLAC), Shanghai, China. We separated all mice randomly into 7 groups (Figure 1) after adaptation: Control, Infected, PB6, PP31, BA11, BA40, BV5. In the Control and Infected group, the mice were orally dosed with 200 μL PBS from day 1 to day 13. The PB6, PP31, BA11, BA40, and BV5 groups were dosed with 200 μL PBS containing 1 × 10^9^ CFU probiotics candidates from day 1 to day 13, respectively. Meanwhile, the mice in Infected, PB6, PP31, BA11, BA40, and BV5 groups were orally challenged with 200 μL resuspension *Clostridium perfringens* (1 × 10^9^ CFU) on day 11, and the mice in Control group was treated with 200 μL PBS. We weighted all mice every day, and mice were free accessed to the water and feed.

### 2.9. Sample Collection and Treatment

After the last gavage and waiting for 12 h, all the mice were weighted and sacrificed. The weights of spleen and liver were recorded as previous study [33]. We used these results to calculate the organ index. We used a vernier caliper to measure the length of colon and collected the blood samples through cardiac puncture. After centrifugation at 3000× *g* (10 min at 4 °C), we obtained the serum. Simultaneously, digesta in the ileum and cecum were collected into 2 mL sterile tubes (Sigma-Aldrich, Los Angeles, CA, USA) for determining the microbiota enumeration.

### 2.10. Bacteria Enumeration of Ileum and Cecum

On day 13, digesta for bacteriological examination were collected aseptically from the ileum and cecum of mice. The population of *Clostridium perfringens*, *Escherichia*, and *Lactobacillus* species in the digesta were detected by the method of absolute quantitative real-time PCR (RT-PCR), as previously described [34], with some modification. Briefly, DNA were isolated from the ileum and cecum. Standard curves for RT-PCR were prepared using DNA extracted from pure cultures to produce a high concentration of the target DNA by normal PCR amplification. Primer sequences were showed in Table 1. We applied *Escherichia coli* DH5α (Takara Bio Inc., Kusatsu, Japan) to generate plasmid standards. We used PCR purification kit (Biomed Gene Technologies, Beijing, China) to purify PCR products and accessed to clone into pCR 2.1 by TA cloning kit (Invitrogen Corporation, Carlsbad, CA, USA). We exerted Nanodrop 2000 (Thermo Fisher Scientific Inc., Waltham, MA, USA) to quantify the purified insert-containing plasmids. Then target DNA copies were calculated [35]:

$$\mathrm{DNA}\;\left(\mathrm{copy}\right)=\frac{6.02\times 10^{23}\;\left(\mathrm{copy}/\mathrm{mol}\right)\times \;\mathrm{DNA}\;\mathrm{amount}\;\left(g\right)}{\mathrm{DNA}\;\mathrm{length}\;\left(\mathrm{dp}\right)\times 660\;\left(g/\mathrm{mol}/\mathrm{dp}\right)}$$

The standard curve was constructed by the ten-fold serial dilutions of plasmid DNA. We used a StepOne Real-Time PCR System (ABI StepOnePlue, Applied Biosystem, Foster City, CA, USA) according to commercial SYBR-Green PCR kit (Takara Biotechnology Inc., Kusatsu, Japan) protocols for absolute qRT-PCR template.

### 2.11. Determination of Inflammatory Cytokines, Immunoglobulin, DAO, and DLA Concentrations

We used ELISA kits (Enzyme-Labeled, Nanjing, Jiangsu, China) to determine Inflammatory cytokines IL-1β, IL-6, TNF-α and immunoglobulin IgA, IgG and fecal sIgA. We detected D-lactate (DLA) and diamine oxidase (DAO) concentrations in serum and the inducible nitric oxide synthase (iNOS) and nitric oxide (NO) in the jejunum using ELISA kits (Enzyme-Labeled, Nanjing, Jiangsu, China). The protocols were followed by previous studies [29,36].

### 2.12. Statistical Analysis

We conducted all experiments three or six times. The results were expressed as mean ± standard deviation (SD). One-factor analysis variance (ANOVA) was used ± to statistical analysis by SPSS 20. A *p*-value < 0.05 was considered to be significant. Graph pad Prism 8 was used to perform a statistical analysis.

## 3. Results

### 3.1. Pathogen Inhibition Using Well Diffusion Assay

**Figure 2:**
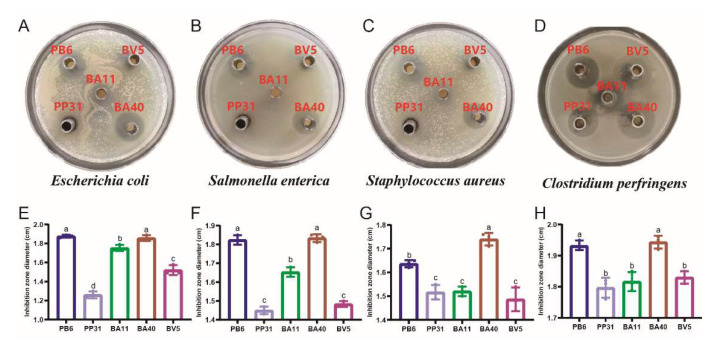
Agar well diffusion assay illustrating the growth inhibition of pathogenic bacteria by cell-free supernatants extracted from the isolated strains. (**A**) *Escherichia coli* plate. (**B**) *Salmonella enterica* plate. (**C**) *Staphylococcus aureus* plate. (**D**) *Clostridium perfringens* plate. (**E**) Inhibition zone diameter of *Escherichia coli* (**F**) Inhibition zone diameter of *Salmonella enterica* (**G**) Inhibition zone diameter of *Staphylococcus aureus* (**H**) Inhibition zone diameter of *Clostridium perfringens*. ^a, b, c^ Means values with dissimilar letters were significantly different (*p* < 0.05). All values contained three repetitions.

The results of antimicrobial activity detection have shown that the isolated strains can inhibit the growth of *Escherichia coli*, *Salmonella enterica*, *Staphylococcus aureus*, and *Clostridium perfringens* (Figure 2A–D). The BA40 presented the best antimicrobial activity against these four foodborne disease microorganisms in humans and animals (Figure 2E–H) than other isolated probiotics (*p* < 0.05). These results indicated that BA40 has the potential as an effective probiotic to resist the pathogens for further studies.

### 3.2. Phenotypic Characteristics of Isolated Strains

**Figure 3:**
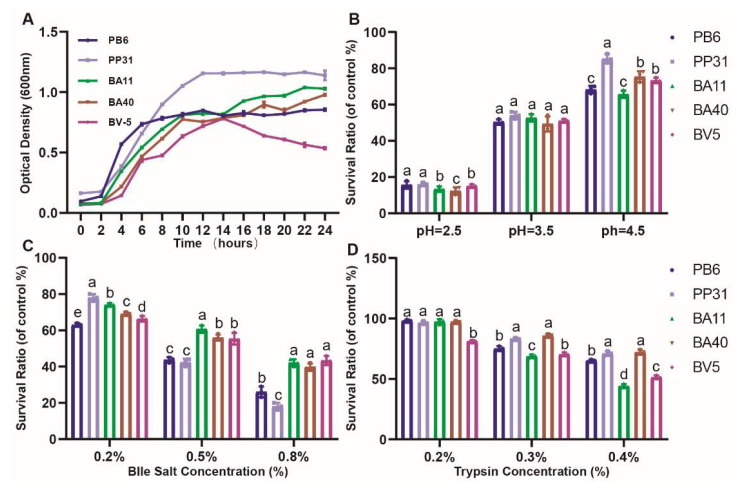
Probiotic properties of isolated strains. (**A**) The growth curves. (**B**) The ability of acid tolerance. (**C**) The ability of bile salt tolerance. (**D**) The ability of trypsin tolerance. ^a, b, c, d^ Means values with dissimilar letters were significantly different (*p* < 0.05). All values contained three repetitions.

Four isolates were selected by agar diffusion assay in Figure 3. The growth curves (Figure 3A) of 4 isolates started to enter the logarithmic growth phase quickly (4 h), and 6–10 h reached the stable phase except PP31 (12 h). The fastest one arrived plateau was strain PB6, which is the positive control; the slowest one was BV5, with a time of 12 h. It is critical for surviving in the gastrointestinal tract to tolerate acid, bile salt and trypsin as probiotics. The isolated strains’ survival rate increased with the rise of pH (Figure 3B). The bile salt and trypsin tolerance test (Figure 3C,D) presented the different results compared to pH assay, because the survival rate of all strains was negatively correlated with the increase in bile or trypsin concentration. There was the highest survival rate in PP31 and PB6 (16.10%, 15.67%) at pH 2.5. BA40 had the best performance in the bile salt and trypsin tolerance test than others (*p* < 0.05), which showed the survival rate of 39.67% at 0.8% bile salt and 72.10% at 0.4% trypsin.

### 3.3. Adhesion of Isolated Strains to IPEC-J2

**Figure 4:**
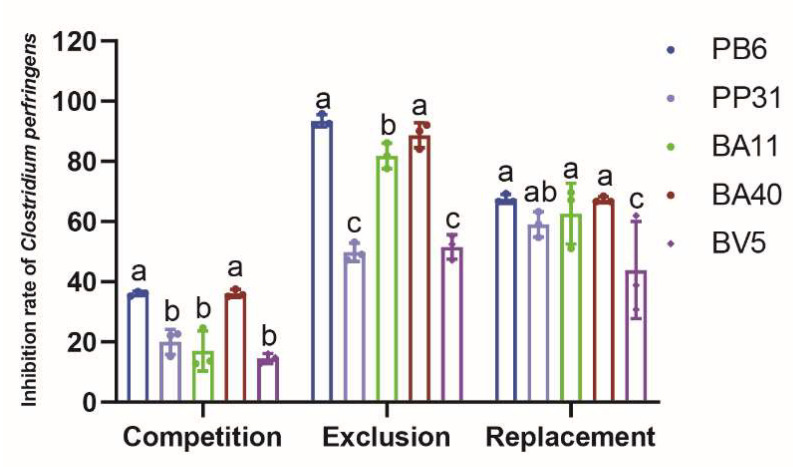
The inhibitory results of isolate strains on the adhesion of *Clostridium perfringens*. ^a, b, c^ Means values with dissimilar letters were significantly different (*p* < 0.05). All values contained three repetitions.

As the data shown in Figure 4, isolate probiotics proved that it inhibited *Clostridium perfringens* adhering to IPEC-J2 cells in exclusion and replacement trails, effectively. In the competition trail, the BA40 had the same ability (36.10%) as PB6 (36.07%) to compete with *Clostridium perfringens*. In the exclusion trail, PB6 (93.47%), BA40 (88.68%), and BA11 (81.84%) presented a stronger ability (*p* < 0.05) to exclude *Clostridium perfringens* than PP31 (49.81%) and BV5 (51.58%). For the replacement experiment, the isolated probiotics had similar results except BV5. The inhibition rate of *Clostridium perfringens* of PB6 (67.52%), PP31 (59.04%), BA11 (62.64%), and BA40 (67.25%) were higher than BV5 (43.85%). All results indicated that the BA40 had an identical ability as PB6 to inhibit the pathogenic bacteria and has the potential to become an antibiotic alternative.

### 3.4. Whole-Genome Sequence of the Isolated Probiotics

Table 2 summarizes the genomic information of these isolated probiotics strains. According to genome sequences obtained using Illumina Hiseq, the genome sequence of PP31, BA11, BA40, and BV5 were presented with genome size, genes on forward strand, genes on reverse strand, rRNA, tRNA, GC content, and GC skew (Supplementary Figure S1A–D). There were several secondary metabolic gene clusters via an antiSMASH analysis, while only four gene clusters harbored 100% similarity in BA11, BA40, and BV5, respectively, to known secondary metabolites (Table 3). The metabolites of these gene clusters were Bacillaene, Macrolactin H, Bacilysin, Bacillibactin, Mersacidin, and Amylocyclicin, with antibacterial, antifungal, antiviral, anti-biofilm, and biocontrol activities. Additionally, we found genes with up to 50% similarity after blasting in the database of virulence factors. We also classified these genes into three groups: defensive virulence factors, offensive virulence factors, and non-specific virulence factors. We found no virulence genes but rather regulatory genes that played important roles in regulating biological processes, including virulence in other bacteria (Supplementary Table S1). Moreover, we found genes > 50% similarity through blasting in the Comprehensive Antibiotic Resistance Database (CARD). There are four genes *ErmB*, *ErmA*, *InuA*, and *poxtA* that are important for the resistance to lincosamide antibiotics, macrolide antibiotics, and streptogramin antibiotics in PP31 with up to 90% similarity (Supplementary Table S2). However, just two genes named *clbA* and *ImrB* are important for the resistance to lincosamide antibiotic phenicol antibiotic in BA11 and BA40 with up to 85% similarity.

**Figure 5:**
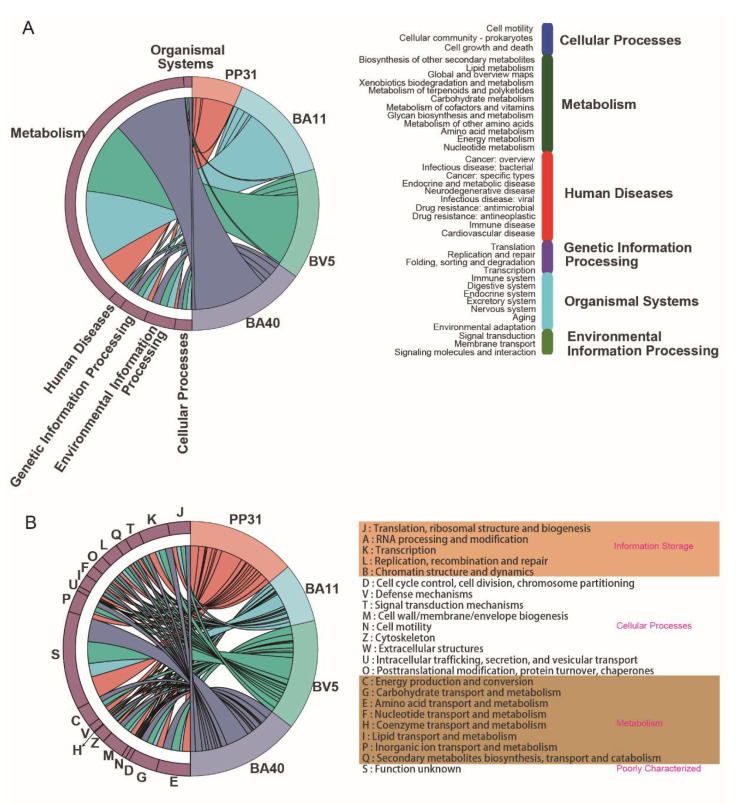
Annotated chord diagrams based on database matching for classification. (**A**) Correspondence between annotated information on bacterial genomes and metabolic pathways obtained by KEGG database. (**B**) The COG database was compared to classify the predicted proteins into gene families and to give the corresponding functional annotation information for the family.

Figure 5 showed the annotated chord diagrams based on KEGG and COG database. BA40 enriched the most genes in first category of KEGG. As the Figure 5A showed, PP31 occupied the minimum genes in KEGG pathway. All isolated probiotics presented the most genes in Carbohydrate metabolism (BA40, 235; BA11, 229; BV5, 226; PP31, 126) which belonged to Metabolism. PP31, BA40 and BV5 were with up 80% of all genes with COG annotation (88.14%, 80.38% and 80.26%). BA40 were annotated the most categories of COG (21) than other isolated probiotics (PP31,19; BA11, 20; BV5, 20) in Figure 5B. E (amino acid transport and metabolism) was the highest abundance annotated in BA40 (289), BA11 (285) and BV5 (284) except S (Function unkonw), while the J (Translation, ribosomal structure and biogenesis) was the highest abundance in PP31 (142).

### 3.5. Effect of Isolated Probiotics Administration on the Mice

**Figure 6:**
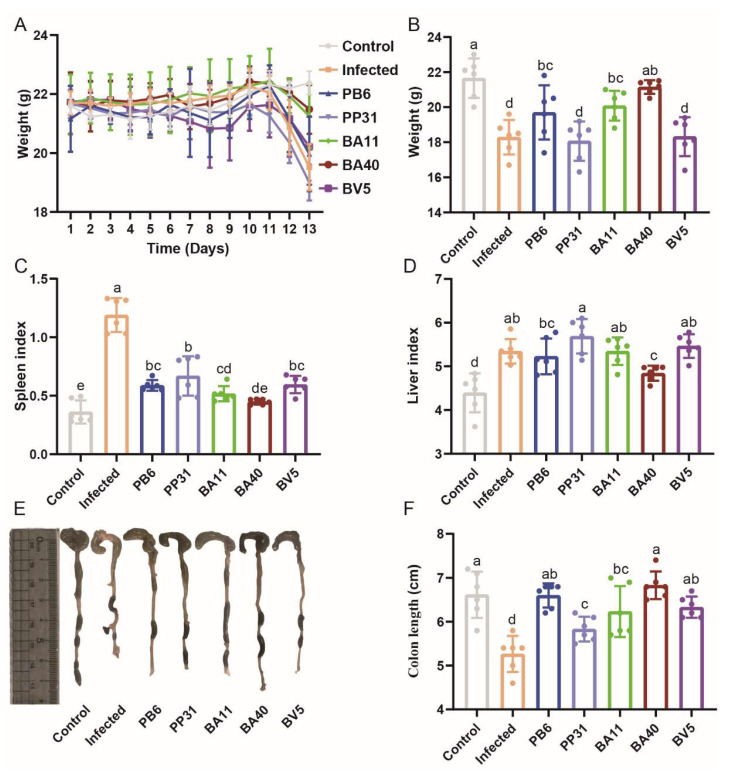
The protective effect of probiotic candidates against *Clostridium perfringens* infection in mice. (**A**) Bodyweight (BW). (**B**) At the end of experiment, mice weight. (**C**) The spleen index. (**D**) The liver index. (**E**) The colon images (**F**) The colon length. ^a, b, c, d^ Means values with dissimilar letters were significantly different (*p* < 0.05). All values contained six repetitions. Partial data of this figure were published in previous study. Adapted with permission from ref. [29]. Copyright 2021 Jiang, Li, Su, Wen, Gong, Zhang, Wang, Jin and Lu.

Figure 6 showed the growth performance of the seven groups during the experiment. Patrial data of this figure were published in our previous study [29]. After being challenged with *Clostridium perfringens,* the body weight (BW) of the Infected group showed a significant decrease (Figure 6A). However, the BW of the BA40 group and the BA11 group almost had no change when compared to the Control group (*p* > 0.05). At the end of the experiment (Figure 6B), the BW of BA40, BA11, and PB6 groups remained unchanged (*p* > 0.05) when compared to the Control group; whereas, the Infected group, PP31 group, and BV5 group showed a dramatic decrease (*p* < 0.05) during the *Clostridium perfringens* treatment. The spleen index and liver index among seven groups were presented in Figure 6C,D. The spleen index of the Infected group showed a significant increase (*p* < 0.05) over other groups. When compared to the Control group, the liver index in the other groups increased markedly (*p* < 0.05). Figure 6E,F showed that the colon length had no difference among the Control, PB6, and BA40 groups (*p* > 0.05). The other six groups showed a significant difference in colon length when compared to the the Infected group (*p* < 0.05). The growth performance indicated that the BA40 could effectively protect against *Clostridium perfringens* infection in mice.

**Figure 7:**
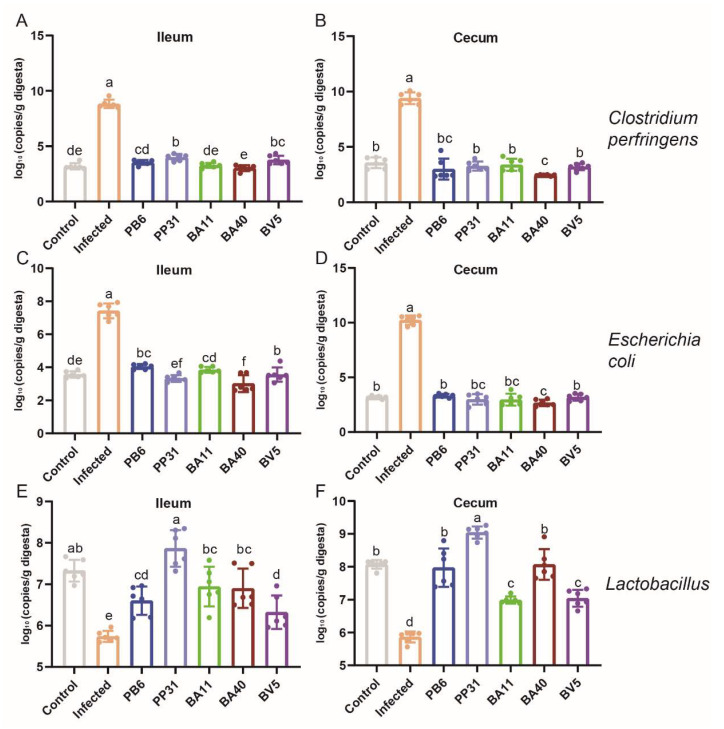
The population of intestinal microbiota of mice. (**A**) *Clostridium perfringens* in the ileum. (**B**) *Clostridium perfringens* in the cecum. (**C**) *Escherichia coli* in the ileum. (**D**) *Escherichia coli* in the cecum. (**E**) *Lactobacillus* in the ileum. (**F**) *Lactobacillus* in the cecum. Results are presented as mean ± SD (The data were presented as log10 gene copies/g of intestinal digesta). ^a, b, c, d^ Means values with dissimilar letters were significantly different (*p* < 0.05).

Figure 7 showed *Clostridium perfringens*, *Escherichia coli,* and *Lactobacillus* enumeration in ileum and cecum in mice. After *Clostridium perfringens* infection in cecum and ileum, the population of *Clostridium perfringens* and *Escherichia coli* increased significantly (*p* < 0.05) when compared to the Control group and other isolated probiotics treatments. The genes copies of *Clostridium perfringens* and *Escherichia coli* decreased remarkably (*p* < 0.05) in BA40. Meanwhile, the population of *Lactobacillus* reduced dramatically (*p* < 0.05) in the Infected group in contrast to the Control group in the ileum and cecum of mice. The *Lactobacillus* slightly increased in the ileum and notably increased (*p* < 0.05) in cecum of mice in the PP31 group. The population of *Lactobacillus* in other groups remained steady or reduced.

**Figure 8:**
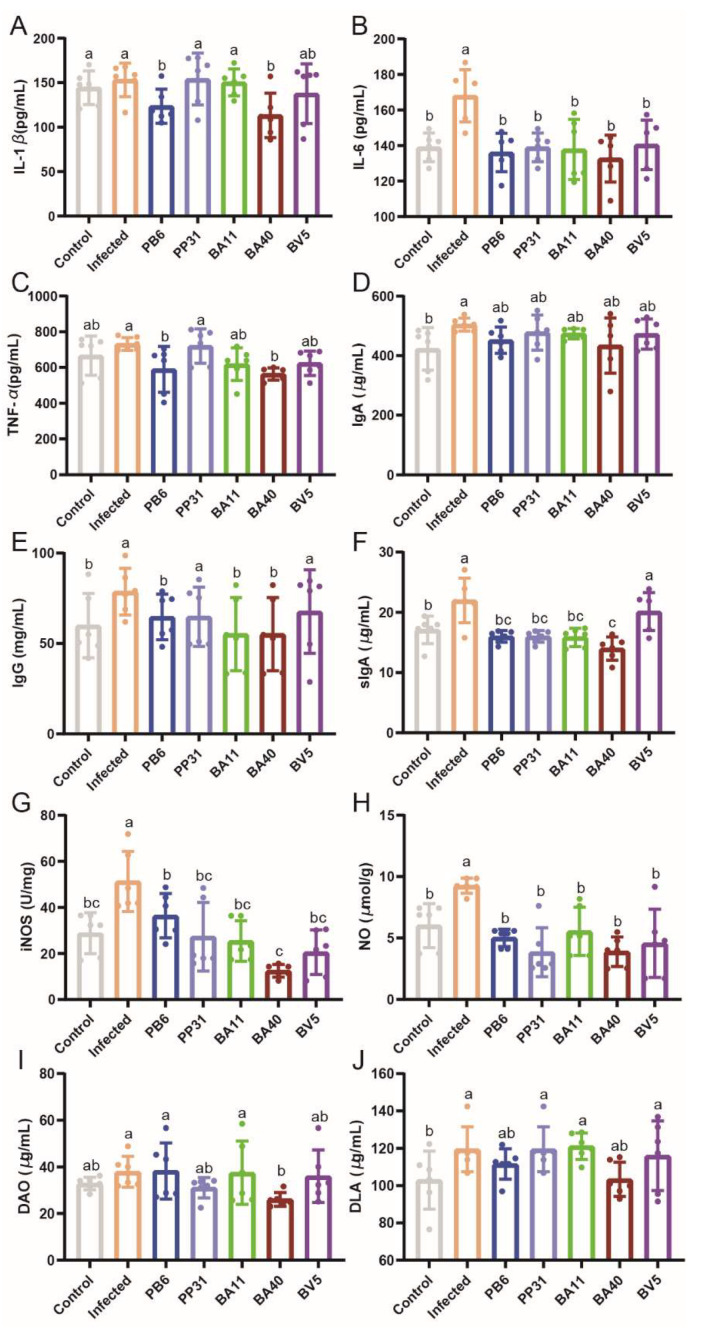
The effect of isolated probiotics treatment on inflammatory cytokines, immunoglobulin, DAO and DLA in mice. (**A**) IL-1β concentrations. (**B**) IL-6 concentrations. (**C**) TNF-α concentrations. (**D**) IgA concentrations. (**E**) IgG concentrations. (**F**) sIgA concentrations. (**G**) iNOS concentrations. (**H**) NO concentrations. (**I**) DAO concentrations. (**J**) DLA concentrations. Results are presented as mean ± SD. ^a, b, c, d^ Means values with dissimilar letters were significantly different (*p* < 0.05). All values contained six repetitions.

The effect of isolated probiotics on the serum inflammatory cytokines, DAO, and DLA were shown in Figure 8. Compared with the Infected group, the IL-1β, IL-6, TNF-α, and IgG (Figure 8A–E) were reduced by BA40 and PB6 treatment (*p* < 0.05), while IgA had no difference (*p* > 0.05). In the Infected group, iNOS, NO concentrations of serum and sIgA (Figure 8F–H) of the jejunum tissue had a significant increase (*p* < 0.05) in contrast to other groups. Figure 8I,J showed the difference of the DAO and DLA concentrations among seven groups. The DLA concentrations were significantly increased (*p* < 0.05) in the the Infected group, and the DAO significantly decreased (*p* < 0.05) by BA40 and pre-treatment compared to the Infected group. The DLA concentrations in the BA40 group (*p* > 0.05) had no difference in contrast to the Control group.

## 4. Discussion

The infectious diseases are caused by pathogenic bacteria (including *Escherichia coli*, *Salmonella enterica*, *Staphylococcus aureus,* and *Clostridium perfringens,* etc.) and are one of the reasons for losses in the husbandry industry [37]. These pathogens are responsible for contracting diseases in humans (foodborne illnesses) and the decline in the livestock production [38,39,40,41]. Antibiotic bans in the farming process are used to reduce problems, such as antibiotic residues and antibiotic resistance [42]. Probiotics are one of the ideal alternatives to antibiotics. Probiotics have been shown to increase the beneficial bacterial in the gastrointestinal tract (GIT), increase nutrient absorption, and feed conversion efficiency and body weight [14,43,44,45,46]. In our study, we aimed to isolate bacterial strains with probiotics properties from the mucosa in JinHua pigs (Chinese panda pig) that could reduce common pathogenic bacteria in pig farms (*Escherichia coli*, *Salmonella enterica*, *Staphylococcus aureus,* and *Clostridium perfringens* etc.). Subsequently, isolated strains were subjected to tolerance trails, antibacterial capacity, adhesion to IPEC-J2, whole genome sequencing (WGS), and in vivo experiments. We revealed that BA40 isolated from JinHua pigs had good probiotic potential.

Given that the ability to inhibit the growth of common pathogenic bacteria on farms is often considered in the selection of potential probiotics to replace antibiotics [42,47,48], we selected four of the most active isolates and further tested their antimicrobial ability. Our antimicrobial well diffusion results revealed that the BA40 showed strong ability in inhibiting pathogens and had the proximate inhibition capacity with PB6. The result were similar with previous studies [49,50,51]. This result suggests that the JinHua pig’s isolates are possibly caused by additional production of antimicrobial compounds. For testing the potential probiotic properties of isolated strains, three trials (including acid, bile salt, and trypsin tolerance) were used to examined their tolerance capacity, because the survival of probiotic bacteria is essential for exerting health benefits and they must remain alive in the GIT to reach the large intestines [52]. Our results showed that *Bacillus* had better growth curve, bile salt, and trypsin tolerance, while *Lactobacillus* had good acid tolerance ability. For further detecting the ability to bind epithelial cell sites of isolates, in the present study, porcine intestinal epithelial cells (IPEC-J2) and the method was used as previous study described [27,53]. The BA40 showed the strong ability to outcompete pathogens for epithelial cell adhesion sites. As we all know, the adhesion to intestinal lining plays an essential role in *Clostridium perfringens* pathogenesis [54,55,56,57]. Our results indicated that BA40 could exclude the *Clostridium perfringens*, and one possible reason is that BA40 has certain adhesion abilities to IPEC-J2. The competition ability was lower than exclusion and replacement ability, which suggested to us that probiotics should be used to prevent the intestinal diseases.

From the WGS results, BA40 had the largest scale of scaffold number and genome size. Bacteriocin genes were identified in isolates strains, suggesting that isolates owned strong pathogen inhibitory activity. Based on the genomic sequencing analysis, BA11, BA40, and BV5 could produce several active compounds such as bacillibactin, marcolactin H, bacilysin, bacillaene, fengycin, and do not contain virulence genes; meanwhile, PP31 only produces coagulin with 40% similarity, and this could illustrate why PP31 had the worst inhibitory activity of pathogenic bacteria among all four isolated strains. Besides, BA11, BA40, and BV5 also possessed different antibacterial abilities, BA40 had the best anti-pathogenic activity. One possible reason that we speculate is the fengycin generated in different quantities (BA11, 20%; BA40, 80%; BV5, 80%). Pipat Piewngam et al. reported that the fengycin secreted by Bacillus could inhibit the pathogens colonized in animal intestines by competitively combining the receptor protein Accessory gene regulator (AgrC) of bacterial quorum-sensing system [58]. Additionally, BA40 is not an antibiotic-resistant bacterium because of susceptibility to the most antibiotics than PP31, BA11, and BV5, including penicillin, cefalexin, ampicillin, streptomycin, kanamycin, gentamicin, ciprofloxacin, chloramphenicol, vancomycin, imipenem, erythromycin, and norfloxacin. Based on KEGG analysis, BA40 were annotated the most genes, 2666 genes, compared other isolates, which were involved in carbohydrate metabolism (8.8%), amino acid metabolism (7.6%), and signal transduction (5.7%) and membrane transport (5.4%). Moreover, there was the same trend with KEGG analysis, the COG analysis annotated the most genes (3177) of BA40 into the most categories (21) than other bacteria. The largest COG group of BA40 was the E (amino acid transport and metabolism), followed by K (transcription), and G (carbohydrate transport and metabolism). Other studies also found that the bacteria possessed potential probiotic ability could enrich these functions based on KEGG and COG database [59,60,61].

As our previous study described, we used the *Clostridium perfringens* to construct a mouse model to test the effect of isolated strains in vivo [35]. We measured isolated strains’ function by analyzing the growth performance, immune status, and the population of beneficial and harmful bacteria. The animal experiment showed that *Clostridium performance* can influence the growth status (including body weight, spleen index, liver index, and colon length) of mice, and BA40 and PB6 were able to attenuate the side effects of *Clostridium perfringens*. In vivo experiments demonstrated that it had the same effect as in vitro experiments. One way the isolated strains exerted the critical role is that it could regulate the gut microbiota of mice. As the reported described, probiotics supplementation could modulate the gut microbiota, improve the immune status, and increase the growth performance. *Lactobacillus* is one of the biomarkers to measure the balance of gut microbial community. However, many harmful bacterial (including *Clostridium perfringens* and *Escherichia coli* etc.) at above normal level also had a negative effect on gut microbiota [1,18,62,63]. Our study results indicated that BA40 had the same ability as PB6 to modulate the balance of gut microbiota, decreasing the population of *Clostridium perfringens* and *Escherichia coli,* and increasing the proliferation of *Lactobacillus*. These results could illustrate how isolated strains to mitigate intestinal microbial disorders by *Clostridium perfringens* challenge. Another possible reason that isolated strains could mitigate the infection of *Clostridium perfringens* is isolates can enhance the inflammatory response by stimulating cytokine production [64]. *Clostridium perfringens* infection induced a strong inflammatory response according to Gong et al. [65] and our results, while isolated strains especially BA40 could reduce the excessive immune response. Meanwhile, BA40 and PB6 also improve the anti-inflammatory cytokine (IL-10, IL-22) concentrations, which can inhibit the immune cells proliferation to decrease the immune response [66,67]. Besides, NO is one biomarker because during the pathogen’s infection process, the immune cells improved pro- IL-1β, TNF-α and INF-γ through promoting NO production [68]. Many studies have revealed that high levels of DAO and DLA can cause intestinal barrier injury or intestinal permeability [69,70]. Our results indicated that BA40 and PB6 can alleviate the intestinal barrier injury.

## 5. Conclusions

Taken together, microbes were isolated from JinHua pigs and screened through a series of assays Then we processed four isolated strains with the highest probiotic potential. Some isolates contained ideal probiotic properties, such as good anti-pathogenic capacity, high survival ratio in acid, bile salt and trypsin environments, lack of virulence, or AMR genes. Notably, we demonstrated that BA40 had antimicrobial activity against enteric pathogens in well diffusion, and we showed its capacity to exclude the attachment of *Clostridium perfringens* to IPEC-J2 cells. The WGS suggested that BA40 had strong antibacterial capacity due to secreting a variety of secondary metabolites at high levels. Besides, the animal experiment illustrated that BA40 and PB6 could reduce the phenomenon induced by *Clostridium perfringens* challenge of body weight loss, colon length decrease, pro-inflammatory cytokine increase, *Clostridium perfringens,* and *Escherichia coli* increase. The present study provides evidence that BA40 could represent a novel probiotic candidate as PB6, which can mitigate the burden of *Clostridium perfringens* associated gut disease or even benefit for human health. Further studies via pig models and clinical studies are required to ascertain the safety and efficacy of JinHua pig-derived probiotics.

## Figures and Tables

**Table 1:** qRT-PCR primers used to quantify intestinal bacteria.

Target	Primer Sequence (5′ to 3′)	Amplicon Size, bp	Reference
*Clostridium perfringens*	F: ATGCAAGTCGAGCGAKG R: TATGCGGTATTAATCTYCCTTT	105	[35]
*Lactobacillus subgroup*	F: AGCAGTAGGGAATCTTCCA R: CACCGCTACACATGGAG	341	[35]
*Escherichia subgroup*	F: GTTAATACCTTTGCTCATTGA R: ACCAGGGTATCTAATCCTGT	340	[35]

**Table 2:** Genomic features of isolated strains.

Strains	16S rRNA Identity	Scaffold Number	Genome Size (bp)	GC %	N50 (bp)	Sequencing Depth (x)	Completeness (%)	Genebank Accession Number
PP31	*Pediococcuspentosaceus*	36	1818617	37.3	260845	659.14	97.3	ON228197
BA11	*Bacillus amyloliquefaciens*	36	3927418	46.42	565069	317.09	99.3	ON227093
BA40	*Bacillus amyloliquefaciens*	143	3969383	46.4	382464	322.08	99.3	ON227058
BV5	*Bacillus velezensis*	36	3867471	46.49	608593	328.7	99.3	ON227128

**Table 3:** Secondary metabolites predicted by the antiSMASH database.

Strains	Cluster Type	MIBiG Accession	Similarity	Location (Start–End)	Gene Number
PP31	Coagulin	BGC0000617	40%	14107–19888	6
BA11	Bacillaene	BGC0001089	100%	142968–252589	52
	Macrolactin H	BGC0000181	100%	471746–559949	44
	Bacilysin	BGC0001184	100%	514804–556223	42
	Bacillibactin	BGC0000309	100%	73512–122977	44
	Difficidin	BGC0000176	53%	131390–177054	31
	Locillomycin	BGC0001005	35%	23265–45904	20
	Fengycin	BGC0001095	20%	1–13205	3
BA40	Bacillibactin	BGC0000309	100%	73491–125283	45
	Bacilysin	BGC0001184	100%	661592–703011	42
	Macrolactin H	BGC0000181	100%	53617–141453	44
	Bacillaene	BGC0001089	100%	360981–470835	52
	Fengycin	BGC0001095	80%	535580–623198	46
	Difficidin	BGC0000176	46%	273837–319624	30
	Plipastatin	BGC0000407	30%	1–13991	12
BV5	Macrolactin H	BGC0000181	100%	507723–595523	44
	Bacilysin	BGC0001184	100%	265403–306822	43
	Mersacidin	BGC0000527	100%	443880–467069	19
	Amylocyclicin	BGC0000616	100%	118402–125992	9
	Fengycin	BGC0001095	80%	1–86692	46
	Bacillaene	BGC0001089	71%	817963–854795	25
	Difficidin	BGC0000176	53%	1–45718	30
	Surfactin	BGC0000433	39%	681718–707084	22

## Data Availability

The whole genome sequence of the four tested strains had been deposited at GeneBank under the BioProject ID PRJNA826263, with accession numbers JALMGL000000000 to JALMGO000000000.

## Supplementary Materials

The following supporting information can be downloaded at: https://www.mdpi.com/article/10.3390/microorganisms10102056/s1, Figure S1: Circos genome circle map of probiotic candidate strains; Table S1: Gene with up to 50% similarity found in probiotics candidate genome according to the database of virulence factors; Table S2: Gene with up to 50% similarity found in probiotics candidates genome according to the Comprehensive Antibiotic Resistance Database (CARD).
