# Supplementary material for: Screening of Bacteria Inhibiting Clostridium perfringens and Assessment of Their Beneficial Effects In Vitro and In Vivo with Whole Genome Sequencing Analysis
Source: Microorganisms. 2022 Oct 18;10(10):2056. doi: 10.3390/microorganisms10102056 (PMC9609858; doi:10.3390/microorganisms10102056)
Supplement: Supplementary file 1 [file microorganisms-10-02056-s001.zip › microorganisms-1945124-up supplementary - Copy/Tabel_S1.pdf]

TABLE S1. Gene with up to 50% similarity found in probiotics candidate genome according to the database of virulence factors

Strains	Gene ID	Level1	Level2	Description	Similarity
	gene0744		Antiphagocytosis	GAS capsular hyaluronate	70.3%
	gene0283		Antiphagocytosis	Prevent phagocytosis	50.5%
	gene0733		Stress protein	Serine protease involved in proteolysis	71.1%
	gene1123	Defensive	Stress protein	Repairs oxidized proteins	63.5%
	gene1265	virulence factors	Antiphagocytosis	Assisting in evading the host immune system	67.9%
PP31	gene1584		Antiphagocytosis	Resistant to complement deposition	58.9%
	gene1594		Stress protein	ATPase	59.8%
	gene1619		Antiphagocytosis	Contributes to host immune evasion	62.7%
	gene0766	Offensive	Adherence	Mediate a complement-independent attachment to host cells	56.4%
	gene1014	virulence factors	Adherence	Surface-expressed elongation factor-Tu	69.2%
	gene1322		Antiphagocytosis	Contributes to host immune evasion	58.2%
	gene1324		Stress protein	Detoxifies H2O2; Protects against ROS	55.9%
	gene1344		Antiphagocytosis	Prevent phagocytosis	51.3%
	gene1345		Stress protein	Serine protease involved in proteolysis	77.9%
	gene1424	Defensive	Antiphagocytosis	Resistant to complement deposition	62.4%
	gene1425	virulence factors	Antiphagocytosis	Contributes to host immune evasion	55.7%
	gene1496		Antiphagocytosis	Assisting in evading the host immune system	70.9%
	gene1640		Stress protein	A cytoplasmic iron superoxide dismutase	52.1%
	gene1862		Antiphagocytosis	Prevent phagocytosis	51.2%
	gene1978		Stress protein	Repairs oxidized proteins	56.6%
	gene2619		Stress protein	ATPase	78.4%
	gene0013		Invasion	YplA, a phospholipase	51.7%
BA11	gene0198		Invasion	Polar flagella required for motility and macrophage invasion	51.3%
	gene0215		Invasion	Penetrating the mucus barrier	52.1%
	gene0216		Secretion system	Translocating different effectors into the host cell,	52.9%
	gene0219	Offensive	Adherence	Glycosylation of the flagellin	50.4%
	gene0227	virulence factors	Adherence	A fibronectin-binding protein	52.2%
	gene0259		Toxin	lysing erythrocytes, polymorphonuclear leukocytes	50%
	gene0260		Secretion system	ESX-1	50.6%
	gene0286		Adherence	Surface-expressed elongation factor-Tu	75%
	gene0305		Adherence	Mediate a complement-independent attachment to host cells	60.1%
	gene0480	Non-	Iron uptake system	Iron uptake	53.8%
	gene0816	specific	Iron uptake system	High-affinity catechol-hydroxamate siderophore	51.0%
	gene0957	virulence factor	Iron uptake system	Catalyzes the formation of tissue-damaging free radicals	54.9%
	gene1048		Iron uptake system	Acquiring iron from transferrin and lactoferrin.Cytotoxic	53.8%
	gene0490		Antiphagocytosis	GAS capsular hyaluronate	58.1%
	gene0365		Antiphagocytosis	Prevent phagocytosis	51.5%
BA40	gene0384		Stress protein	Serine protease involved in proteolysis	77.9%
%	gene0489		Antiphagocytosis	Resistant to complement deposition	62.1%
	gene1303		Antiphagocytosis	Contributes to host immune evasion;	59.1%
	gene2008	Defensive	Antiphagocytosis	Prevent phagocytosis	50.4%
	gene2024	virulence factors	Stress protein	Repairs oxidized proteins	56.6%

	gene2262		Stress protein	Detoxifies H2O2; Protects against ROS	55.9%
	gene2877		Stress protein	A cytoplasmic iron superoxide dismutase	52.1%
	gene2994		Antiphagocytosis	Assisting in evading the host immune system	70.9%
	gene3571		Stress protein	ATPase	78.4%
	gene1282		Invasion	YplA, a phospholipase	51.7%
	gene1215		Adherence	A fibronectin-binding protein	52.2%
	gene1241		Adherence	glycosylation of the flagellin	50.4%
	gene1274	Offensive	Secretion system	Translocating different effectors into the host cell	52.9%
	gene1285	virulence factors	Invasion	Penetrating the mucus barrier	52.5%
	gene1286		Invasion	Polar flagella required for motility and macrophage invasion	51.3%
	gene3551		Adherence	Mediate a complement-independent attachment to host cells	60.2%
	gene3597		Adherence	Surface-expressed elongation factor-Tu mediates attachment	74.7%
	gene0100		Iron uptake system	catalyzes the formation of tissue-damaging free radicals	55.1%
	gene0204	Non-	Iron uptake system	Iron uptake	53.4%
	gene2767	specific	Iron uptake system	High-affinity catechol-hydroxamate siderophore	50.6%
	gene3790	virulence factor	Iron uptake system	Acquiring iron from transferrin and lactoferrin.Cytotoxic	66.7%
	gene3888		Iron uptake system	May be necessary for penetration to deeper tissues	54.9%
	gene1647		Antiphagocytosis	GAS capsular hyaluronate	56.7%
	gene0015		Stress protein	Detoxifies H2O2; Protects against ROS	55.9%
	gene0763		Antiphagocytosis	Contributes to host immune evasion;	58.2%
	gene0844		Antiphagocytosis	Assisting in evading the host immune system	70.9%
	gene0961	Defensive	Stress protein	A cytoplasmic iron superoxide dismutase	52.1%
	gene1646	virulence factors	Antiphagocytosis	Resistant to complement deposition	65%
	gene1648		Antiphagocytosis	Contributes to host immune evasion;	55.3%
	gene2391		Stress protein	Repairs oxidized proteins	56.6%
	gene2754		Antiphagocytosis	Prevent phagocytosis	51.5%
	gene2777		Stress protein	Serine protease involved in proteolysis	77.9%
	gene3575		Stress protein	ATPase	78.4%
BV5	gene0742		Invasion	YplA, a phospholipase	51.7%
	gene0675		Adherence	A fibronectin-binding protein present on the listerial surface	52.2%
	gene0701		Adherence	glycosylation of the flagellin	50.4%
	gene0734	Offensive	Secretion system	Translocating different effectors into the host cell	52.1%
	gene0745	virulence factors	Invasion	Penetrating the mucus barrier	52.1%
	gene0746		Invasion	Polar flagella required for motility and macrophage invasion	51.3%
	gene1819		Toxin	Lysing erythrocytes, polymorphonuclear leukocytes	50%
	gene2938		Adherence	Mediate a complement-independent attachment to host cells	60.2%
	gene3549		Adherence	Surface-expressed elongation factor-Tu mediates attachment	75%
	gene1519	Non-	Iron uptake system	High-affinity catechol-hydroxamate siderophore	51%
	gene2593	Specific	Iron uptake system	Iron uptake	53.8%
	gene3287	virulence factor	Iron uptake system	catalyzes the formation of tissue-damaging free radicals	54.8%
