# Supplementary material for: Screening of Bacteria Inhibiting Clostridium perfringens and Assessment of Their Beneficial Effects In Vitro and In Vivo with Whole Genome Sequencing Analysis
Source: Microorganisms. 2022 Oct 18;10(10):2056. doi: 10.3390/microorganisms10102056 (PMC9609858; doi:10.3390/microorganisms10102056)
Supplement: Supplementary file 1 [file microorganisms-10-02056-s001.zip › microorganisms-1945124-up supplementary - Copy/Table_S2.pdf]

TABLE S2 Gene with up to 50% similarity found in probiotics candidates genome according to the Comprehensive Antibiotic Resistance Database (CARD)

Strains	Gene ID	*ARO name	Drug class	Similarity
	gene0264	msbA	nitroimidazole antibiotic	57.4%
	gene0578	rpoB2	peptide antibiotic; rifamycin antibiotic	59.7%
	gene1266	arlR	acridine dye; fluoroquinolone antibiotic	54.7%
PP31	gene1710	ErmB	lincosamide antibiotic; macrolide antibiotic; streptogramin antibiotic	98.8%
	gene1714	ErmA	lincosamide antibiotic; macrolide antibiotic; streptogramin antibiotic	90.6%
	gene1728	fexA	phenicol antibiotic	58.4%
	gene1886	lnuA	lincosamide antibiotic	97.5%
	gene1888	poxtA	lincosamide antibiotic;macrolide antibiotic; oxazolidinone antibiotic	99.6%
	gene0099	rphB	rifamycin antibiotic	78.9%
	gene0536	ykkD	aminoglycoside antibiotic; phenicol antibiotic; tetracycline antibiotic	81.7%
	gene0537	ykkC	aminoglycoside antibiotic; phenicol antibiotic; tetracycline antibiotic	79.5%
	gene0547	tetA	penam; tetracycline antibiotic	52.5%
	gene0658	Bla1	penam	64.4%
	gene0744	FosB	fosfomycin	62%
	gene0786	blt	acridine dye;fluoroquinolone antibiotic	77.8%
	gene2055	tet(L)	tetracycline antibiotic	79.9%
	gene2056	SAT-4	nucleoside antibiotic	52.2%
BA11	gene2460	vmlR	lincosamide antibiotic; macrolide antibiotic; oxazolidinone antibiotic	71%
	gene2480	clbA	lincosamide antibiotic; phenicol antibiotic; pleuromutilin antibiotic	97.4%
	gene2870	dfrG	diaminopyrimidine antibiotic	51.9%
	gene3106	tmrB	nucleoside antibiotic	76.6%
	gene3146	lmrB	lincosamide antibiotic	88.4%
	gene3160	mphK	macrolide antibiotic	65.8%
	gene3269	aadK	aminoglycoside antibiotic	63.4%
	gene3464	mprF	peptide antibiotic	78.9%
	gene3567	rpoB2	peptide antibiotic; rifamycin antibiotic	64.3%
	gene3693	ampC1	cephalosporin; penam	52.5%
	gene1409	rphB	rifamycin antibiotic	79.3%
	gene1885	SAT-4	nucleoside antibiotic	52.2%
	gene1886	tet(L)	tetracycline antibiotic	80.7%
	gene2035	dfrG	diaminopyrimidine antibiotic	52.5%
	gene2428	blt	acridine dye; fluoroquinolone antibiotic	77.8%
	gene2472	FosB	fosfomycin	62%
	gene2543	BcII	cephalosporin; penam	53.1%
	gene2592	vmlR	lincosamide antibiotic; macrolide antibiotic; oxazolidinone antibiotic	71.2%
	gene2614	clbA	lincosamide antibiotic;phenicol antibiotic; pleuromutilin antibiotic	99.4%
	gene3021	ampC1	cephalosporin;penam	53.3%
BA40	gene3106	lmrB	lincosamide antibiotic	88.6%
	gene3148	tmrB	nucleoside antibiotic	76.6%
	gene3179	aadK	aminoglycoside antibiotic	63.4%
	gene3270	bcrA	peptide antibiotic	79.1%

	gene3271	bcrB	peptide antibiotic	55.6%
	gene3272	bcrC	peptide antibiotic	57.6%
	gene3307	mprF	peptide antibiotic	78.4%
	gene3343	Bla1	penam	64.1%
	gene3452	tetA	penam;tetracycline antibiotic	52.5%
	gene3462	ykkC	aminoglycoside antibiotic;phenicol antibiotic;tetracycline antibiotic	81.5%
	gene3463	ykkD	aminoglycoside antibiotic;phenicol antibiotic;tetracycline antibiotic	81.7%
	gene3591	rpoB2	peptide antibiotic;rifamycin antibiotic	64.3%
	gene0184	blt	acridine dye;fluoroquinolone antibiotic	77.6%
	gene0228	FosB	fosfomycin	62.8%
	gene0299	BcII	cephalosporin;penam	51.1%
	gene0316	Bla1	penam	64.7%
	gene0412	tetA	penam;tetracycline antibiotic	52.5%
	gene0422	ykkC	aminoglycoside antibiotic;phenicol antibiotic;tetracycline antibiotic	80.4%
	gene0423	ykkD	aminoglycoside antibiotic;phenicol antibiotic;tetracycline antibiotic	82.7%
	gene1048	tet(L)	tetracycline antibiotic	80.5%
	gene1049	SAT-4	nucleoside antibiotic	51.7%
BV5	gene2380	dfrG	diaminopyrimidine antibiotic	52.5%
	gene2885	rphB	rifamycin antibiotic	78.9%
	gene3016	aadK	aminoglycoside antibiotic	63%
	gene3092	tmrB	nucleoside antibiotic	77.7%
	gene3132	lmrB	lincosamide antibiotic	88.4%
	gene3146	mphK	macrolide antibiotic	64.1%
	gene3339	mprF	peptide antibiotic	78.9%
	gene3451	vmlR	lincosamide antibiotic; macrolide antibiotic; oxazolidinone antibiotic	71%
	gene3555	rpoB2	peptide antibiotic; rifamycin antibiotic	64.3%
	gene3653	ampC1	cephalosporin;penam	52.8%

*ARO means Antibiotic Resistance Ontology
