# Supplementary figures and images for: Screening of Bacteria Inhibiting Clostridium perfringens and Assessment of Their Beneficial Effects In Vitro and In Vivo with Whole Genome Sequencing Analysis
Source: Microorganisms. 2022 Oct 18;10(10):2056. doi: 10.3390/microorganisms10102056 (PMC9609858; doi:10.3390/microorganisms10102056)

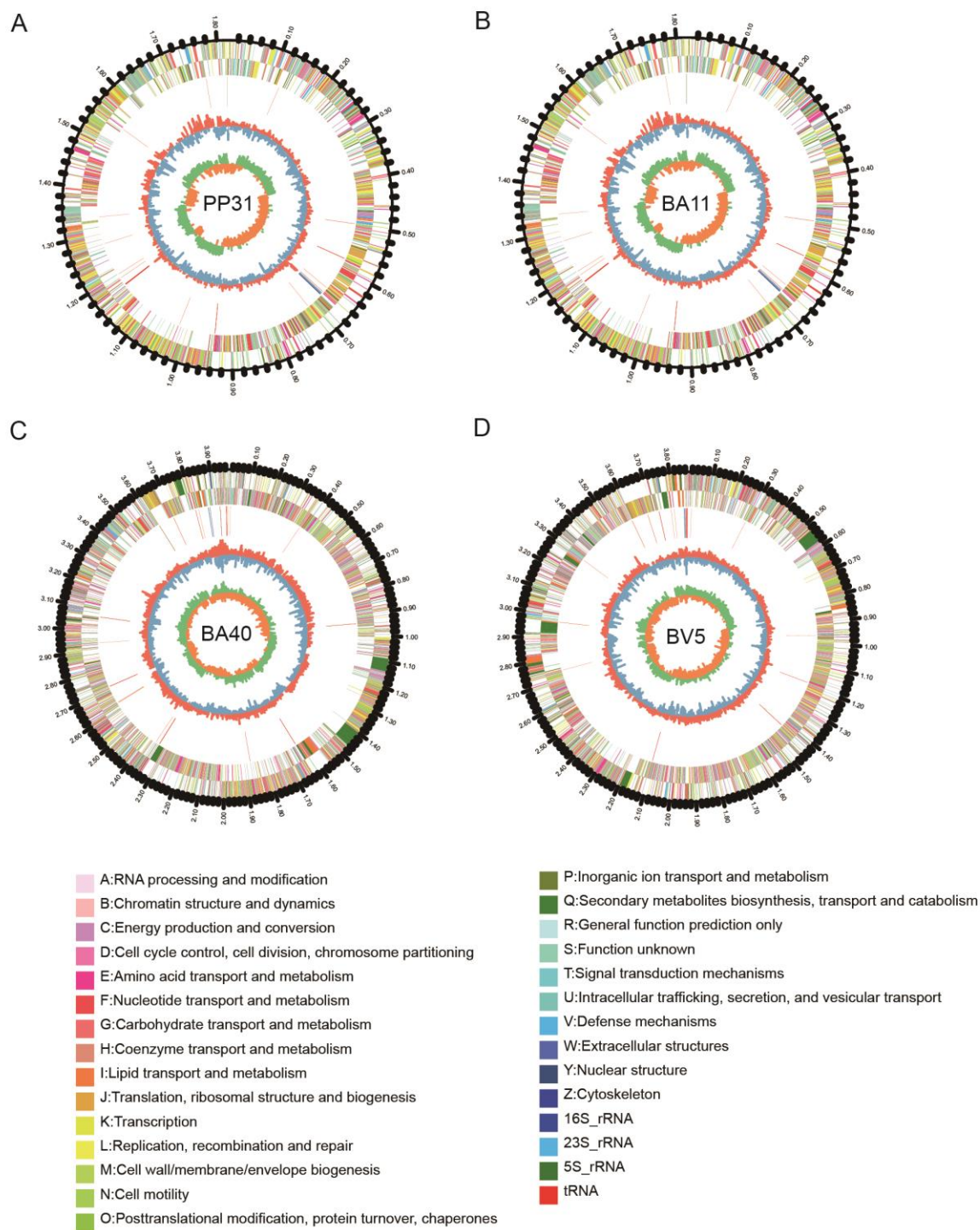

Figure S1: Circos genome circle map of probiotic candidate strains

Supplement: Supplementary file 1 [file microorganisms-10-02056-s001.zip › microorganisms-1945124-up supplementary - Copy/Figure S1.pdf]

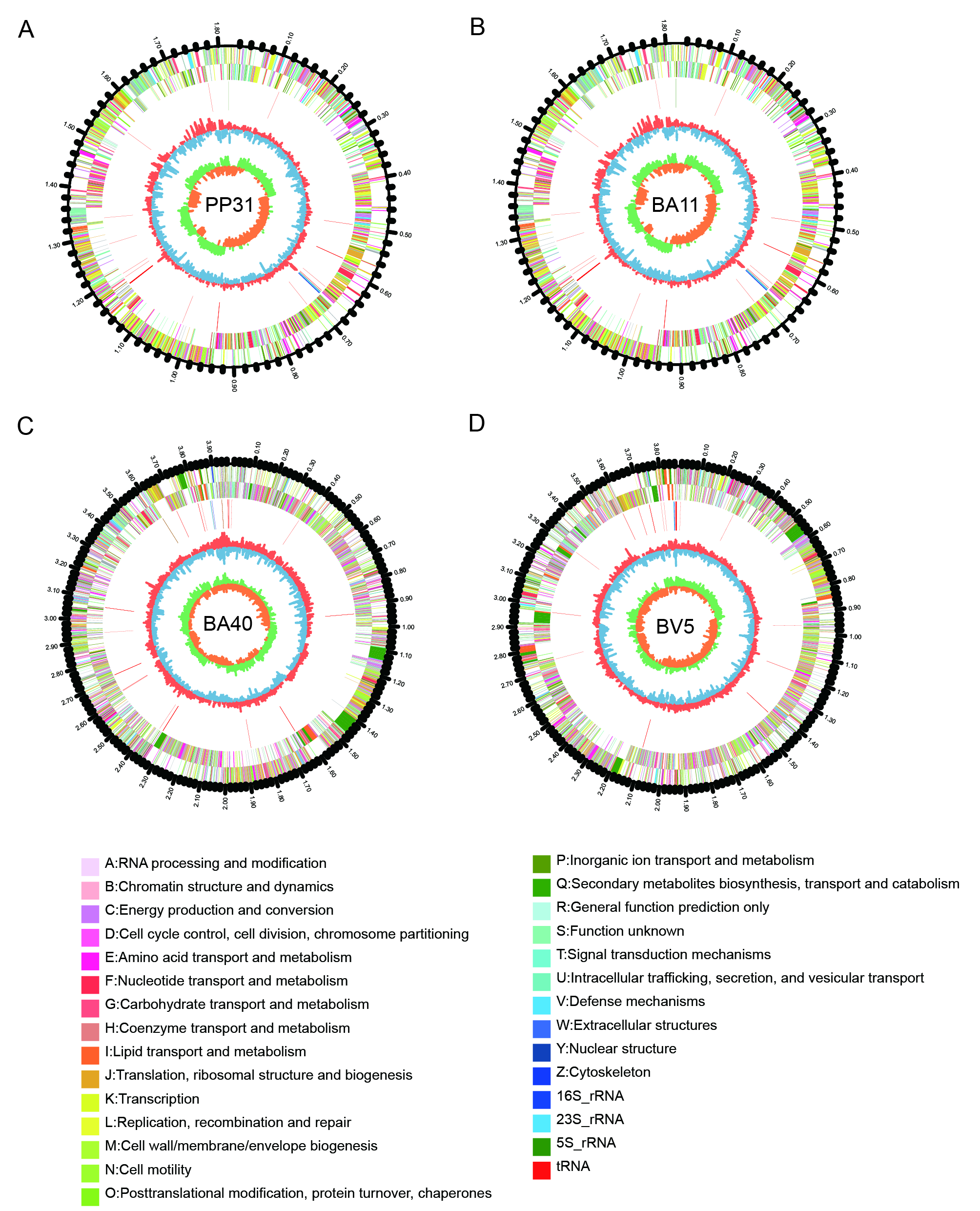

Supplement: Supplementary file 1 [file microorganisms-10-02056-s001.zip › microorganisms-1945124-up supplementary - Copy/FigureS1.tif]
